# Source of Sustained Voltage Difference between the Xylem of a Potted *Ficus benjamina* Tree and Its Soil

**DOI:** 10.1371/journal.pone.0002963

**Published:** 2008-08-13

**Authors:** Christopher J. Love, Shuguang Zhang, Andreas Mershin

**Affiliations:** Center for Biomedical Engineering, Massachusetts Institute of Technology, Cambridge, Massachusetts, United States of America; University of Melbourne, Australia

## Abstract

It has long been known that there is a sustained electrical potential (voltage) difference between the xylem of many plants and their surrounding soil, but the mechanism behind this voltage has remained controversial. After eliminating any extraneous capacitive or inductive couplings and ground-mediated electric current flows, we have measured sustained differences of 50–200 mV between the xylem region of a Faraday-caged, intact, potted *Ficus benjamina* tree and its soil, as well as between its cut branches and soils and ionic solutions standardized to various pH values. Using identical platinum electrodes, no correlation between the voltage and time of day, illumination, sap flow, electrode elevation, or ionic composition of soil was found, suggesting no direct connection to simple dissimilar-metal redox reactions or transpirational activity. Instead, a clear relationship between the voltage polarity and magnitude and the pH difference between xylem and soil was observed. We attribute these sustained voltages to a biological concentration cell likely set up by the homeostatic mechanisms of the tree. Potential applications of this finding are briefly explored.

## Introduction

We were intrigued by Internet circulated reports of sustained voltage differences of around 1 V between aluminum nails inserted into tree trunks and copper electrodes planted into the adjacent soil [1]. We immediately suspected a dissimilar-metal redox reaction was taking place with the tree-soil system acting as a giant electrolyte reservoir (similar to a Galvanic “potato” battery), so we carried out our measurements using identical platinum electrodes at both ends. Even so, we continued to measure between 50 and 200 mV of sustained voltage several hours and days after electrode insertion.

Upon closer examination of the relevant literature it became apparent that an electrical potential difference (voltage) between parts of trees, including phloem [2], xylem [3], and leaves [4] and between such parts and the adjacent soil had been routinely observed and reported for decades but the origin of this voltage remains controversial and a subject of considerable debate [5]–[7].

These voltage differences have been used in attempts to monitor plant activity and have been hypothesized to be due to various sources, most prominent of which appears to be the “streaming potential” mechanism [7], which is itself related to transpiration and sap flow.

Here we postulate a simpler hypothesis: the sustained voltage difference routinely observed between parts of trees and soil is mainly due to a difference in pH between the two. Specifically, the tree-root-soil system acts as a concentration pH cell, sometimes actively maintained by the tree's homeostasis mechanisms. The potential from such a concentration cell is the Nernst potential, which only depends on a concentration gradient. At equilibrium (no net ionic flux across the interface), the Nernst potential is equal to the diffusion potential that results from charge separation across a permeable interface by diffusion down a concentration gradient [8].

## Results

We tested this hypothesis by systematically measuring the voltages between the xylem of a potted *Ficus benjamina* tree and soils of various pH that showed a clear correlation closely following the Nernst equation. We controled for possible external sources of voltage such as radio-frequency-noise pickup, telluric (ground) currents [9], and dissimilar-metal redox reactions at the electrodes. Additionally, we calculated that no more than 20% and likely less than 1% of the magnitude of the voltages measured can be attributed to a streaming potential mechanism.

**Figure 1:**
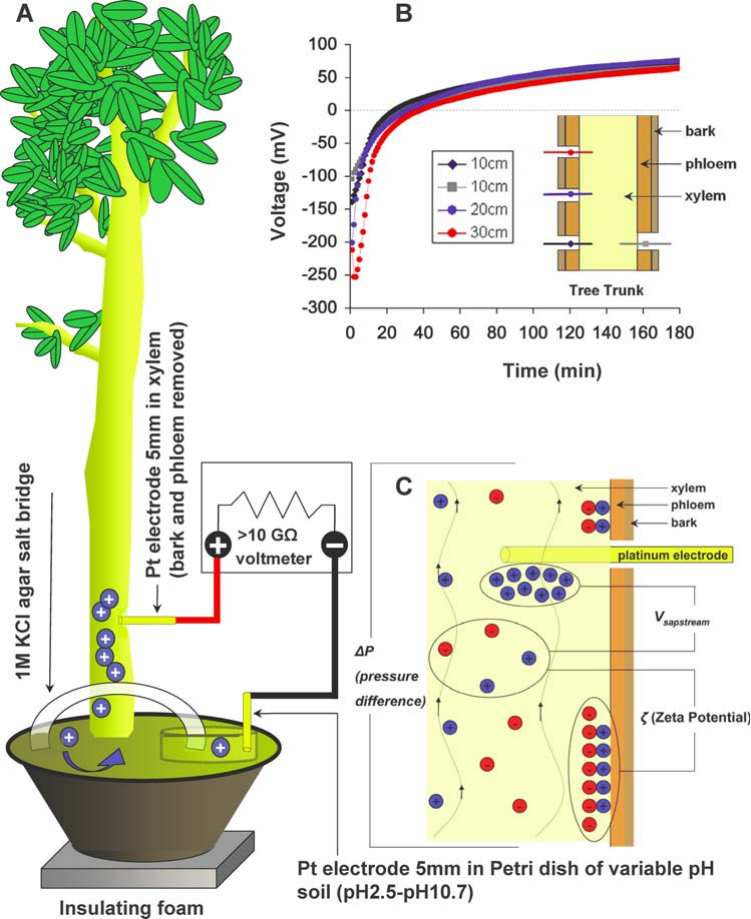
Experimental Setup and the Streaming Potential. A A potted *Ficus benjamina* was placed on insulating foam inside a Faraday cage. Identical Platinum electrodes where inserted into the xylem (phloem removed) and a Petri dish containing a standardized water content soil solution of variable pH. The electrodes were connected to a high-impedance voltmeter. The standardized soil was connected to the pot soil via a 1 M KCl agar salt bridge (to complete the circuit via the soil-root interface). B Voltage vs Time post-electrode insertion shows no dependence on height, orientation or sap flow (it was stopped by inserting razor blades above and below the electrode) once transient voltages and currents are allowed to dissipate. The difference in pH between the xylem and the soil in this case is ∼2. C The “streaming potential” voltage generation mechanism depends on the Zeta potential (*ζ*) -voltage difference due to different flow properties at the center of a capillary and its walls and the *ΔP* (pressure difference between the two ends of the capillary and is given by 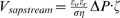 which, for typical values for a tree, yields between 1 and 10 mV. *V_sapstream_* is such that faster flow leads to higher voltages.

Fig. 1A shows the voltage differences between the xylem and the soil of an indoor potted *Ficus benjamina* tree measured at one-minute intervals over a ten-hour period. To exclude the possibility of radio-frequency noise pickup, we placed the tree and apparatus in a Faraday cage but we soon determined that the voltages measured outside the cage were within experimental error to the voltages recorded inside, leading us to conclude that whatever environmental electromagnetic noise present in our laboratory would not significantly affect our measurements. The instantaneous current under short circuit conditions was found to be less than 1 µA, depended weakly on soil water content and was most likely regulated by electrode-soil interfacial resistance.

We observed no significant change in the electrical potential difference between the xylem of the trunk region of the tree and soil with changing height or cardinal orientation of electrode placement around the tree (Fig. 1B, and consistent with previous reports [5]). We determined that variations in sap flow also played no detectable role in the magnitude or polarity of the sustained voltage since we mechanically stopped all flow in one experiment by inserting razor blades above and below the length of the electrode and by using severed branches in other experiments. Furthermore, transpiration and sap flow in our potted *Ficus benjamina* was minimal because the tree was placed indoors and exposed to regular sunlight through a glass window, not a sunlamp. Therefore, the bulk of the voltage could not be due to ions flowing past the electrode (which is the mechanism behind “streaming potential” -depicted in Fig. 1C) and thus not directly related to transpiration.

Instead, we found that the voltage between xylem and soil is approximately constant up, down, and around the tree, consistent with a mechanism depending on the approximately constant pH (around 6) throughout the xylem in the measured areas.

**Figure 2:**
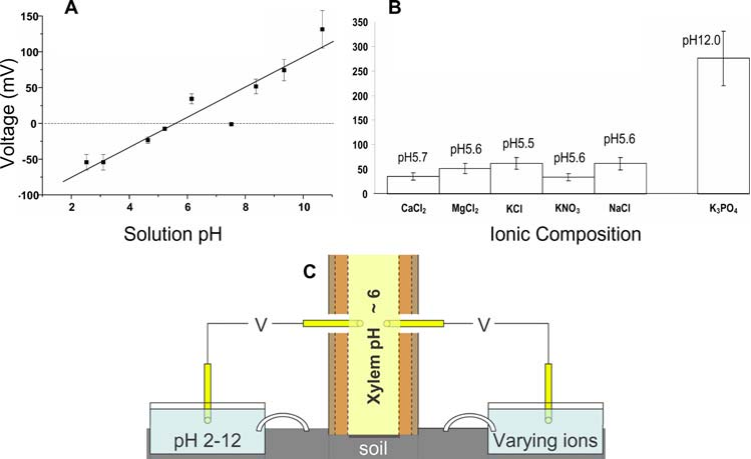
Varying pH and Ionic Composition. A The effect of varying pH on the voltage between xylem (which is at constant pH∼6) and a standardized ionic solution connected to the pot soil via a 1 M KCl agar salt bridge is consistent with a mechanism governed by the Nernst equation. B To explore possible redox chemistry effects, the ionic composition of the solutions was varied (while keeping pH constant) but virtually no effect on the observed voltage between xylem and solution. K_3_PO_4_, of pH 12 gave a voltage consistent with a roughly 60 mV step per unit of pH difference, as predicted by the Nernst equation. C The experimental set up for A and B above (the experiments where performed separately).

**Figure 3:**
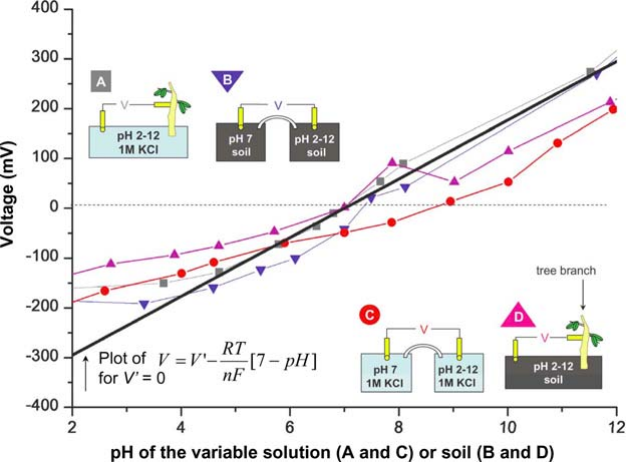
Comparing Voltages to the Nernst Equation. To further test our hypothesis that the voltages between xylem and soil are due to a biological concentration cell as governed by the Nernst equation, we plotted the voltage vs variable pH between a cut branch (no significant transpiration) and ionic solutions (A –grey squares) and soil solutions (D –pink triangles). To confirm the suitability of our salt bridges we also plotted the voltages between a standard pH 7 soil solution and variable pH soils (B –blue triangles) and a standard (pH 7) and variable ionic solutions (C –red squares). In all cases a roughly 60 mV per step of pH mismatch was evident and plots intersected the zero y-axis between pH 6 and 7 as expected. A plot of the Nernst equation with zero residual voltage *V′* and a reference of pH 7 is included (black solid line).

We carefully calibrated our apparatus using in vitro pH differences in soil and 1 M KCl solutions, and we compared these to values measured between a fresh tree branch and soil at manipulated pH values. Fig. 2A shows a clear trend of increasing voltage between the xylem and an ionic liquid of increasing pH values. Fig. 2B indicates that the ionic composition does not significantly affect the voltage observed as long as the pH difference is kept constant. In both cases, the voltage magnitudes and polarities are consistent with a voltage generation mechanism dependent on unequal concentration of ions given by the Nernst equation (Fig 3).

## Discussion

### Mechanism behind sustained voltage

A large portion of the relevant literature incorrectly assumes the origin of voltage differences between xylem and soil are the result of a “streaming potential” (Fig 1C) due to the flow of ion-containing sap past the electrode [8], [10]–[12]. We have shown that eliminating sap flow does not appreciably change the voltage difference observed, meaning its bulk must originate elsewhere. The streaming potential, *V_sapstream_* is given by: 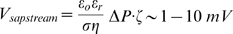 where *ε_o_* = dielectric permeability of vacuum = 8.85×10^−12^F/m (C^2^/J m); and, in the case of our Ficus, *ε_r_* represents the dielectric constant of xylem (∼80); *σ* is its typical conductivity (∼0.01 S/m ); and *η* the viscosity (∼10^−3^ Pa s); Δ*P* the pressure difference (∼1 MPa) and *ζ* the “Zeta potential” (0.01 V) due to the difference in mobility between liquid and pore wall atoms.

The order-of-magnitude estimate for the streaming potential for conditions typical of trees as above yields a value between 1 mV and 10 mV, which does not account for the entire 50–200 mV we routinely observed. In addition, the streaming potential mechanism for voltage difference generation is such that faster flow makes for larger voltage differences–which is inconsistent with published observations that show an exactly opposite relationship [12], where it is clear that the highest voltages are recorded during the least transpirational activity (and therefore slowest flow) and vice versa. In addition to correctly predicting the trends and magnitudes of the measured voltages between xylem and soil, our proposed mechanism accounts for the variability of voltage between different soils and tree types as well as the observed differences between the same type tree growing in different pH soils. The small alternating fluctuations with periods in the 12 hour range as reported by other groups [5], [12], [13] may still be related to transpiration effects but are unlikely to be due to a streaming potential related mechanism because of the exactly negative correlation they exhibit in some of these reports [12], [13]. According to our measurements, the bulk of the voltage follows a difference in pH rather than any more intricate effects.

We have determined that in contrast to a streaming potential mechanism, the Nernst equation, governing a pH concentration cell voltage generation mechanism closely predicts our observed measurements (Fig 3): 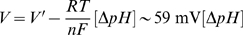 where *R* is the universal gas constant = 8.314 J K^−1^ mol^−1^, *T* is the temperature in degrees Kelvin, *F* is the electronic charge times Avogadro's number (Faraday's constant) = 9.648×10^4^C mol^−1^ and [Δ*pH*] is the difference in pH between two reservoirs. While there is active discussion on the validity of Kleiber's metabolic-rate *h* to mass M scaling “law” [14] in plants [15] it still stands as the lower limit [16] so we can estimate the minimum metabolic rate *h* of our Ficus tree (of mass M∼5 kgr) to be 200 kcal per day (a power output of about 10 W). The voltages recorded, multiplied by an estimated average short circuit current *I*∼0.1–1 µA indicate a drawn electrical power (*P* = *IV*) of between 5 and 200 nW that is, our circuitry parasitically harvested no more than 2×10^−6^% of the tree's power and its presence is unlikely to be developmentally detrimental at least from a purely metabolic standpoint.

The form of the Nernst equation used above is representative of an electromotive force when there is no current flow and *V′*, the cell membrane potential, is assumed near zero because the xylem tissue is mainly composed of dead cells and, our electrode due to its large size relative to cell dimensions, pierces many cellular walls and essentially averages any residual membrane potentials.

Negligible current was drawn by the measurement circuit through the electrode–xylem, electrode–soil, and root–soil interfaces meaning that the voltage values we report include the over-potential needed to drive actual electron flow up from the soil to the xylem. Lowering the impedance (as, for instance, to use the xylem-soil potential difference to drive a load resistor, charge a battery, etc) will necessarily lower the available voltage by the value equal to the over-potential (in favor of a small current *I*).

### Applications

Measurements of plant tissue pH using traditional methods such as Litmus paper or electronic pH meters are inaccurate, difficult, and sometimes destructive as they involve the extrusion of large amounts of sap or excision of tissue. Our work suggests that measuring the voltage difference between parts of plants and parts of plants and known-pH solutions using minimally destructive microelectrodes can be used to monitor changes in pH inside plant tissue and therefore many metabolic and other pH-sensitive processes.

We found it difficult to resist speculating that there may be possible practical applications of these findings beyond monitoring pH changes such as a wide variety of trickle chargers for niche, low-power, pulsed, off-grid distributed systems–including forest fire detectors; environmental sensors; and “smart dust” or mesh-networked devices drastically decreasing the need for in-the-field battery changes. Interestingly, ionic flows through microfluidic circuits have already been investigated as viable sources of microwatt level electrical power [18] via the streaming potential effect (in the case of trees and other plants we can expect between 1–10 mV from sap flow alone as discussed above). If a method for easily inserting low-impedance microelectrodes in high-flow areas was developed the sap flow could be converted into electrical power. In addition, the voltage generated by the mismatch in pH between xylem and soil can also be harvested by such circuits that would act as a low-impact “parasite” on the tree drawing on its metabolism, assuming the homeostatic mechanisms would continue supplying the puncture site with redox-mediating molecules.

Such devices must necessarily draw some current, deplete redox mediators at the positive-electrode site, and lead to some negative-electrode degeneration over long times. However, it is possible, by correctly choosing a bio-friendly positive-electrode plating material (e.g. graphite, platinum, gold etc.), to harness large plants' metabolic power and drive tiny load resistors.

## Materials and Methods

### Noise control

The Faraday cage was a vibration-isolated basement room surrounded on all sides, including floor and ceiling, by solid sheet copper, designed to eliminate all electromagnetic interference (such as cosmic radiation, radio waves, lightning and power line noise) from high fidelity scanning tunneling microscopy experiments. Our potted Ficus was placed on insulating foam and all equipment was properly grounded. All measurements were carried out using platinum electrodes connected to a high input impedance (Z>10 GΩ) data-logging multimeter (Keithley 2000, Keithley Instruments Inc., Cleveland, Ohio). A custom-made holder ensured reproducible placement and minimal movement of the electrodes during experimentation (Fig. 1A). The negative terminal of the voltmeter was attached to the varying pH soil, ion solution, or 1 M KCl varying pH solution, and the positive terminal to the tree's xylem or a reference ionic solution (Fig. 1A, Fig. 2C, Fig. 3).

### Electrodes and voltage measurements

Electrodes were made of pure platinum (0.5 mm diameter, ESPI, Ashland, Oregon). We note that platinum electrodes are not ideal for this type of voltage measurement because of the semiliquid phase of the xylem and soil, but the alternatives (calomel and Ag-AgCl), were impractical in our case due to their large size and form factors. Before each set of experiments, the platinum electrodes were polished with nylon pads containing ∼50 nm alumina micropolish (Buchler, Lake Bluff, IL), rinsed with deionized water, and thoroughly wiped with a Kimwipe after each individual experiment. The electrode surface area in contact with the active volume was constant at both sites at approximately 15 mm^2^. Phloem was removed from all measurement sites to avoid averaging effects due to possibly dissimilar pH and/or sap flow direction.

### Solutions and pH measurements

All ionic and soil solutions were based on doubly-filtered water from a Millipore Simplicity (model SIMS 60000). Standardized soil samples of identical water content were prepared by adding 2 g of potting soil (Fafard, Agawam, MA) to 20 ml of water, mixing thoroughly, and adjusting the pH with KOH or HCl. To standardize ionic solutions at a particular pH, 1 M KCl was used to increase the availability of diffusible current carriers, increase the solution's conductivity and avoid unpoised electrodes [18] (except in the case of Fig. 2B where we specifically used other ions to test for any redox chemistry effects). The pH of the ionic solutions in Fig. 2B was again adjusted with KOH or HCl. We determined xylem pH by measuring liquid exudates collected with a needle from the stem of a branch whose leaves were subjected to high pressure in a pressure bomb. Enough liquid was collected to record a pH value of approximately 6 on a pH strip (Corning, MA), and the value was calibrated against a glass-electrode pH meter (Orion Model 310, Thermo Scientific, Waltham, MA). To obtain a value for our standard pH calibration graph (Fig 3), 20 ml of a 1 M KCl solution at a particular pH was added to a Petri dish. A second Petri dish contained 20 ml of a 1 M KCl solution at the reference pH 7 and these were connected by a salt bridge consisting of 1 M KCl in agar gel.
